# Self‐reported immune status and COVID‐19 associated subjective cognitive functioning in post‐COVID‐19 syndrome: Examination of an Irish cohort

**DOI:** 10.1002/brb3.70027

**Published:** 2024-09-24

**Authors:** Jessica Holland, Sinead Brown, Susan O'Flanagan, Stefano Savinelli, Kathleen McCann, Keith Gaynor, Patrick Mallon, Eoin Feeney, Grace Kenny, Christine Boyd, Fiadhnait O'Keeffe, Jessica Bramham

**Affiliations:** ^1^ School of Psychology University College Dublin Dublin Ireland; ^2^ Department of Psychology St Vincent's University Hospital Dublin Ireland; ^3^ Department of Infectious Diseases St Vincent's University Hospital Dublin Ireland

**Keywords:** COVID‐19, cognition, depression, immune response, Post‐Covid syndrome

## Abstract

**Introduction:**

Cognitive changes are very frequently reported by people with post‐COVID‐19 syndrome (PCS), but there is limited understanding of the underpinning mechanisms leading to these difficulties. It is possible that cognitive difficulties are related to immune status and/or low mood. The aim of the present study was to examine the relationship between immune status and cognitive functioning in PCS, while considering whether depression symptoms also influence this association.

**Methods:**

Participants were recruited in an online study of cognitive and psychological consequences of PCS, involving individuals attending a post‐COVID clinic in an acute general hospital in Ireland, and a comparison sample of age‐ and sex‐matched community controls who had also been infected with COVID‐19 but had not experienced PCS. Participants with PCS (*n* = 71) and community controls (*n* = 50) completed the immune status questionnaire, Cognitive Failures Questionnaire (CFQ), Hospital Anxiety and Depression Scale (HADS), and the Functional Assessment of Chronic Illness Therapy‐Fatigue Scale (FACIT‐F).

**Results:**

Significant differences were observed between groups in terms of perceived immune status, perceived cognitive function, depression scores, and fatigue, with the “PCS” group reporting lower immune status, more cognitive difficulties, and higher levels of depression and fatigue. Regression analysis in the PCS group indicated that immune status and depression significantly contributed to variance in subjective cognitive functioning, with immune status remaining a significant predictor of cognitive functioning scores even when accounting for depression, fatigue, and other covariates related to PCS, such as Body Mass Index (BMI).

**Conclusion:**

Our findings suggest that subjective cognitive functioning is influenced by self‐reported immune status in PCS, emphasising the importance of immune status, cognitive, and mood screening as part of routine clinical care in PCS.

## Graphical abstract

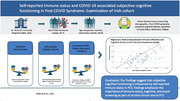

## 1 INTRODUCTION

Post‐COVID‐19 syndrome (PCS) is defined as a heterogeneous condition with persistent clinical signs and symptoms that appear following COVID‐19 infection, lasting for more than 12 weeks, not better explained by an alternative diagnosis (Carod‐Artal, 2021). With over 200 symptoms of PCS documented (Davis et al., 2023), some common complaints include fatigue, shortness of breath, cognitive dysfunction, and complaints generally have an impact on everyday functioning (WHO, 2021). While the underlying biological mechanisms of PCS are unknown, low‐grade inflammation has been associated both with low mood and cognitive difficulties prior to the COVID‐19 pandemic (Rosenblat et al., 2015; Zalli et al., 2016), adding to mounting evidence that suggests an abnormal or excessive autoimmune and inflammatory response may play an important role in PCS.

After recovery from infection in SARS and MERS, impairment of memory, attention, concentration, and processing speed were reported in more than 15% of patients (Hopkins et al., 1999; Sheng et al., 2005). Similarly, a phenomenon described as “COVID‐19 brain fog” following COVID‐19 infection has been reported, including memory deficits, impaired executive functioning, and attention difficulties at several weeks, and even months post‐discharge (Almeria et al., 2020). A meta‐analysis of 43 studies found that 17%–28% of patients either report or exhibit cognitive impairment at least 3 months following COVID‐19 diagnosis (Ceban et al., 2021), with cognitive difficulties often changing the daily lives of those affected (Ferrucci et al., 2021; Mazza et al., 2021). These impairments can be severe even in individuals with no previous neurological conditions and can lead to a dysexecutive syndrome described by a third of people recovered from severe illness (Helms et al., 2020). Moreover, individuals with cognitive complaints have higher scores in anxiety and depression (Almeria et al., 2020; Méndez et al., 2022), and those who experience cognitive difficulties from COVID‐19 are at a higher risk for developing neurological diseases (Heneka et al., 2020) adding to the disease burden of PCS (Ferrucci et al., 2021; Mazza et al., 2020; Mazza et al., 2021; Taquet et al., 2021).

Substantial literature has demonstrated that inflammation can severely disrupt brain function, with theories suggesting aberrant immune response or a “cytokine storm” underpins neurocognitive impairments in PCS (Bower et al., 2022). Accordingly, several inflammatory markers and cytokines have been found to be elevated in COVID‐19 patients, including C‐reactive protein (CRP), interleukin (IL)−1β, IL‐2 receptor, interferon‐γ, and tumor necrosis factor‐α (TNF‐α) (Hartung et al., 2022; Hu et al., 2021; C. Huang et al., 2021; Lier et al., 2022; Poletti et al., 2021; Zhou et al., 2020). Furthermore, studies have found that performance on neuropsychological testing is linked with inflammation among individuals with PCS even months after disease recovery, such that those who perform more poorly on cognitive tests have elevated inflammatory markers 12 weeks or more following infection (de Paula et al., 2022; Evans et al., 2021; Ferrando et al., 2022; Mazza et al., 2021). However, due to variation in the timing of testing and in cognitive tests used, more generalized research is needed to examine the relationship between cognition and inflammation in this population.

As substantial overlap exists between presentations in depression and PCS, and symptoms of depression contribute to disability and need for care in those with PCS (Ferrucci et al., 2021; Mazza et al., 2020; Mazza et al., 2021; Taquet et al., 2021), further exploration of the link between immune response, perceived cognition and depression is warranted. The aim of this study is to examine the relationship between immune status and cognitive difficulties in PCS, while exploring whether any observed relationship was attributable to variation in depression. Using an online questionnaire, we quantified immune status, cognitive difficulties, and depressive symptoms based on self‐report measures. Data were collected from people who had been infected with COVID‐19 seeking clinical care for PCS (PCS group) and age‐ and sex‐matched controls who did not report PCS in the community. Other factors associated with impairment in PCS were also explored in relation to cognitive function, namely, fatigue, as this is the most common symptom in PCS (Ceban et al., 2022), as well as BMI, sleep, and biological sex, as these factors have been found to be significant predictors of PCS symptoms (Ceban et al., 2022). Based on these data, the following hypotheses were explored: (1) Individuals with PCS will experience differences in immune status and low mood when compared to community controls (CC). (2) Individuals with PCS will report increased cognitive difficulties, which will be associated with differences in immune response, as measured by self‐reported immune status. (3) The relationship between immune status and subjective cognitive functioning will remain significant irrespective of depression scores and remain significant when covariates are added to the model (BMI, biological sex, fatigue, and sleep).

## 2 METHODS

### 2.1 Participants

Participants (*N* = 121) were recruited from an online study of cognitive and psychological consequences of PCS, involving individuals attending a post‐COVID‐19 clinic in an acute general hospital, and a comparison sample of age‐ and sex‐matched CC who had also been infected with COVID‐19. All participants had self‐reported positive tests for COVID‐19 at the time of infection, with most participants tested at home, using PCR or antigen testing kits. Ethical approval for the study was obtained from St Vincent's University Hospital Research Ethics Committee in December 2021, with PCS sample recruitment carried out in early 2022.

The initial PCS sample included 91 participants who attended the “Long Covid clinic” for various complaints related to PCS symptomatology between March 2022 and February 2023. The PCS sample was recruited for the study by clinicians in the hospital “Long Covid” clinic. To ascertain diagnosis, the WHO case definition of PCS was used, which includes symptoms of fatigue, shortness of breath and changes in cognitive functioning (WHO, 2021). Within the PCS sample, 20 individuals were excluded due to incomplete responses, pre‐existing neurological disorders, and to mitigate the effects of aging on perceived cognitive function, participants aged above 65 were also excluded. For the remaining *N* = 71 individuals who formed the “PCS group,” six had moderate COVID‐19 (conventional hospitalization without mechanical ventilation) in the acute phase and three had severe symptoms requiring mechanical ventilation. For participants that were hospitalized at the time of infection, COVID‐19 was confirmed using PCR. All others (*N* = 62) were not hospitalized during acute COVID‐19 infection but had been deemed eligible for the study based on their presentation and medical examination at the PCS clinic.

For the CC sample, 188 individuals were initially recruited via an online survey advertised by University College Dublin, open to anyone interested in taking part in the study between November 2022 and January 2023. Participants were excluded if they had a history of head injury, if they had pre‐existing neurological conditions or if they were aged over 65. As most people had been infected with COVID‐19 by the time of data collection, the control sample was ascertained using the Post‐Covid‐19 Functional Scale (PCFS), and all those who scored 3 and above were excluded from the sample. A higher score on the PCFS scale would suggest that control participants are experiencing post‐COVID‐19 symptoms, not suitable for comparison with the PCS group. The final “CC group” consisted of *N* = 50 individuals after age and sex matching.

Most of the CC group (*N* = 39) reported PCFS grade 0, that is, no limitations in my everyday life and no symptoms, pain, depression, or anxiety related to the infection, and *N* = 11 reported PCFS grade 1, that is, negligible limitations in my everyday life and negligible symptoms of pain, depression, or anxiety related to the infection. The PCS group, who have all been seen by a medical doctor in a hospital clinic specifically for Long COVID, reported more long‐lasting consequences following COVID‐19 infection, as displayed in distribution of their PCFS scores: two individuals reported a score of 0 on the PCFS scale, 10 individuals scored 1 on the PCFS scale, while 29 individuals in this group reported a PCFS score of 2 and 30 individuals reported a PCFS score of 3.

## 3 MEASURES

Participants completed an online survey and were given an option to return to the survey later to reduce the effects of fatigue on questionnaire responses.

### 3.1 Demographics questionnaire

The survey demographics section assessed age, sex, ethnicity, BMI, and the date of a positive COVID PCR/antigen test. Individuals were also asked whether they had any previous history of psychological diagnosis, such as anxiety or depression, and information on other health conditions.

### 3.2 Post Covid Functional Status scale

The PCFS, developed by Klok and colleagues (2020), is a self‐report instrument, designed to evaluate the consequences of COVID‐19 and their impact on functional status. It is used to assess a range of functional limitations, such as changes to lifestyle and physical activity. The impact on usual duties is assessed in five scale grades, from grade 0 (no functional limitations) to grade 4 (severe functional limitations) (see Appendix). The scale included questions such as *“Do you suffer from symptoms, pain, depression, or anxiety?,” or “Can you live alone without any assistance from another person?”*. Since its creation in 2020, the PCFS scale has been validated in highly symptomatic adult subjects with confirmed and presumed COVID‐19, 3 months after the onset of symptoms (Machado et al., 2021).

### 3.3 Immune status questionnaire

The ISQ aims to assess perceived immune status over the preceding year (Wilod Versprille et al., 2019). The ISQ consists of seven items, including “common cold,” “diarrhea,” “sudden high fever,” “headache,” “muscle and joint pain,” “skin problems (e.g., acne and eczema),” and “coughing”. All items were scored on a 5‐point Likert scale stating how often participants experienced these complaints over the past 12 months, with “never,” “sometimes,” “regularly,” “often,” and “(almost) always” as answering possibilities. The overall ISQ score ranges from 0 to 28, with higher scores indicating more challenges to the immune system. A score of 8 or above indicates “poor” immune status (Wilod Versprille et al., 2019). The ISQ has appropriate face, content, and construct validity and is a reliable, stable, and valid method to assess the past 12 month's perceived immune status, used in COVID‐19 populations (Huang et al., 2023). Previous research with the ISQ reported a Cronbach's *α* of 0.632 and a test‐retest reliability of 0.80 (Wilod Versprille et al., 2019), with a Cronbach's *α* of 0.75 in the current study. This questionnaire has also been validated in English speaking populations (Wilod Versprille et al., 2019)

### 3.4 Cognitive failures questionnaire

The cognitive failures questionnaire (CFQ) is a 25‐item self‐report measure of cognitive failures in everyday life. Developed by Broadbent et al., the cognitive failures questionnaire measures the frequency with which people experience cognitive difficulties in everyday life, such as absent‐mindedness, slips and errors of perception, memory, and motor functioning (Broadbent et al., 1982). Participants respond on a 5‐point scale ranging from never to always. It is scored from 0 to 100 with a high score indicating an increased tendency to experience cognitive failures. The CFQ has been used in COVID‐19 populations (Miskowiak et al., 2021), with findings of previous studies indicating good internal consistency and test‐retest reliability in the general population (Bridger et al., 2013). Correspondingly, Cronbach's α in the current study was 0.96 for this scale.

### 3.5 Hospital anxiety and depression scale

The HADS is a brief 14‐item self‐report measure consisting of two subscales, each with 7 items, assessing levels of anxiety and depression, respectively. Each item is rated on a four‐point scale (e.g., 0 = not at all; 1 = from time to time, occasionally; 2 = a lot of the time; 3 = most of the time). A score of 0–7 indicates normal functioning, with 8–10 being indicative of borderline clinical cases, and scores of greater than 11 indicating clinical levels of depression or anxiety. For this study, a cut‐off score of ≥8 points was used for each scale since this value has shown good sensitivity and specificity to determine the presence of anxiety or depression (Olssøn et al., 2005). The HADS is considered an appropriate measure for assessing the severity of anxiety and depression with hospitalized patients with COVID‐19 (Fernández‐de‐Las‐Peñas et al., 2022), with Cronbach's *α* values 0.89 for the anxiety scale (HADS‐A) and 0.85 for the depression scale (HADS‐D) reflecting the internal consistency reliability in a post‐COVID‐19 population (Fernández‐de‐Las‐Peñas et al., 2022). Cronbach's *α* in the current study was 0.88 for the HADS depression scale and 0.90 for the HADS anxiety scale. A binary score for depression is computed, using clinical cut‐off values (HADS depression scores ≥8).

### 3.6 Functional Assessment of Chronic Illness Therapy‐Fatigue (FACIT‐F) scale

The FACIT‐F is a short, 13‐item tool that measures an individual's level of fatigue during their usual daily activities over the past week, and is a widely used and validated self‐report questionnaire to assess fatigue symptoms (Cella et al., 2002; Montan et al., 2018; Piper & Cella, 2010; Yellen et al., 1997). The level of fatigue is measured on a four‐point Likert scale (4 = not at all fatigued to 0 = very much fatigued), and a sum score ranging from 0 (worst fatigue) to 52 (no fatigue) is calculated. A binary score for fatigue is computed using clinical cut‐off values (FACIT‐F scores ≥30). Clinically relevant fatigue was defined by scores ≤30, as suggested by the creators of the scale, based on general population data (Piper & Cella, 2010). This scale has been used in studies of COVID‐19 populations (Hartung et al., 2022), with a Cronbach's *α* value of 0.95 in the current study reflecting internal consistency.

## 4 ANALYSIS

First, groups were compared on demographics and the key variables of subjective immune status, subjective cognitive function, and mood, as well as factors associated with PCS such as fatigue and BMI. *T*‐tests were carried out in SPSS (version 27) to assess for group differences in ISQ scores, CFQ scores, HADS depression scores, FACIT‐F scores, and BMI using linear variables and scaled scores were possible. Furthermore, a chi‐squared test was carried out to access for group differences in history of psychiatric illness.

To test the associations between immune status (independent variable) and cognitive functioning (dependent variable), linear regressions were carried out in SPSS in the whole sample, with an interaction term created for group × ISQ score, and then in the PCS sample only following results of analysis. An a priori power analysis was conducted using G*Power version 3.1.9.7 (Faul et al., 2007) to determine the minimum sample size required to test the study hypothesis using regression with two predictor variables. Results indicated the required sample size to achieve 80% power in regression detecting a medium effect size of 0.15, at a significance criterion of *α* = .05, was *N* = 68. Thus, the obtained sample size of *n* = 71 in the PCS group is adequate to test the study hypothesis. As a mediating role of depression cannot be observed due to temporality of variables examined, a binary score for depression was added as a covariate in the regression model, with depression classified as HADS depression scores ≥8. This is to test whether inclusion of depression in the model changes the relationship between immune status and cognition. Due to their potential impact on cognitive function and PCS symptoms, BMI, sex, and fatigue were also included in analysis as covariates. Fatigue was made into a binary score to use as a covariate in analysis, according to FACIT‐F scale classifications (fatigue classified by scores ≥30).

Covariates were included in the model using a stepwise approach, that is, model 1: no covariates, model 2: BMI and sex; model 3: BMI, sex, and fatigue (binary); and model 4: BMI, sex, fatigue (binary), and depression (binary).

## 5 RESULTS

For each regression analysis, assumptions of linear regression were checked using P–P plots and scatter plots. To test for an association between immune status and cognitive functioning, linear regression was carried out on the whole sample and then analysis on the PCS group only, based on the four models described in the methods section. All predictor variables yielded a variance inflation factor (VIF) of <1.4, indicating some small or moderate correlations between predictor variables in the whole sample, not assumed to affect results (see Table 1). In order to reduce multi‐collinearity observed between independent variables, HADs anxiety was removed and a HADs depression binary score was used in analysis only.

**TABLE 1:** Relationships between predictor variables, correlation and VIF.

	1. ISQ	2. FACIT‐F	3. HADS‐D	
	Correlation coefficients			Regression VIF
1. ISQ	1			1.21
2. FACIT‐F	.35*	1		1.33
3. HADS‐D	.26*	.37*	1	1.21

Abbreviations: FACIT‐F, functional assessment of chronic illness therapy‐fatigue scale (binary); HADS‐D, hospital anxiety and depression scale, depression score (binary); ISQ, immune status questionnaire; VIF, variance inflation factor.

Statistically significant results at *p = .05 level.

## 6 DESCRIPTIVE STATISTICS

Descriptive information for all variables analyzed is shown in Table 2. As per the WHO definition of post‐COVID‐19 syndrome, differences were observed between groups as regards reported cognitive difficulties and fatigue (see Table 2). Cognitive failures questionnaire (CFQ) scores were significantly higher in the PCS group (*t* = 7.32, *p* ≤ .001), and fatigue scores as measured by the FACIT‐F were significantly higher in the PCS group (*t *= 11.18, *p* ≤ .001). These effects remained significant when correcting for the number of between group differences tests (Bonferroni corrected *p*‐value = .05/4 = .012). A significant difference in BMI was detected between groups (*t* = 2.42, p = .017), however, this result did not remain significant with Bonferroni correction. Groups did not differ as regards history of anxiety (*X* ^2^(1, *N* = 122 = 1.09, *p* = .29), or history of depression (*X* ^2^(1, *N* = 122 = 2.22, *p* = .14).

**TABLE 2:** Descriptive statistics for the whole sample, *N* = 121.

	PCS *N* = 71	CC *N* = 50	Coefficient	*p*‐value
	M (SD)	M (SD)	*t*	
Age, years	46.94 (9.70)	43.06 (13.18)	1.77	.80
Sex (male:female)	16:55	14:36	.47*	.49
CFQ	50.22 (20.23)	25.88 (16.23)	7.33	<.001
ISQ	8.97 (4.84)	4.36 (3.65)	5.97	<.001
HADS‐D	9.5 (4.56)	3.5 (3.63)	8.04	<.001
FACIT‐F	30.75 (10.31)	10.52 (9.42)	11.18	<.001
BMI	26.74 (5.46)	24.70 (3.85)	2.42	.17
MH history (yes)	22	16	.14*	.91
				
Mean time since COVID‐19 (in weeks)	86	25		
Participants who were hospitalized	9	0		
Mechanical ventilation	3	0		
PCFS scale				
Grade 0	2	39		
Grade 1	10	11		
Grade 2	29	0		
Grade 3	30	0		
				

Abbreviations: BMI, body mass index; CC, community control group; CFQ, cognitive failures questionnaire; FACIT‐F, functional assessment of chronic illness therapy‐fatigue scale; HADS‐D, hospital anxiety and depression scale, depression score (binary); ISQ, immune status questionnaire; MH, mental health; PCFS, post covid functional status scale; PCS, post‐COVID syndrome group.

*Chi‐squared test used, *X* ^2^ statistic.

There was no significant difference between the groups regarding age or sex as the samples were chosen to be matched on these variables. As there was discrepancy in time of infection between the two groups due to nature of data collection, and vaccine was not available at the time of infection for some participants. More information on vaccine availability COVID‐19 variants likely to have caused illness are provided in table (see Table 3). Of note, a large proportion of the PCS group are healthcare workers. Furthermore, the majority were infected during the first wave of COVID‐19 in Ireland, with original (“alpha”) COVID‐19 variants most common, between December 2019 and July 2021.

1. Individuals with PCS will experience changes in immune status and mood when compared to community controls.

Using *t*‐tests to compare mean values, significant differences between the PCS group and community controls were found for ISQ scores (*t* = 5.97, *p* ≤ .001), HADS depression scores (*t* = 8.04, *p* ≤ .001), with the PCS group having higher levels of symptoms on both measures.

2. Individuals with PCS will report increased cognitive difficulties, which will be influenced by compromised immune response as measured by self‐reported immune status.

Using regression analysis in the whole sample, an interaction term was created to analyze the effect of group and immune status on CFQ scores. This analysis found that interaction effects were significant (*b* = −2.25, *p* = .049), where those in the PCS group with higher ISQ scores scored lower on the CFQ scale. The effect of immune status on cognition was then examined in the PCS group only, as this is the primary interest of the study.
To further examine the relationship between immune status and cognitive functioning PCS, regression analysis was carried out in the PCS group only (*N *= 71). In this analysis, the effect of ISQ on CFQ further was found to be significant (*b* = −.552, *p* ≤ .001, *R* = .305). When including BMI and sex in the model (covariates included due to their relationship with immune response) relationship between immune status and cognitive functioning remained significant (*b* = −.548, *p* = .001, R2 change = .018).

3. The relationship between immune status and subjective cognitive functioning will remain significant irrespective of the presence of a depression score in the model (as measured by binary variable according to clinical cut‐off values) and will not differ when covariates are added to the model (BMI, sex, or binary fatigue variable).

**TABLE 3:** Further sample characteristics related to COVID‐19 variants and vaccination availability in an Irish Context.

		CC	PCS	Total
Variant at the time of infection	Alpha	12	50	62
	Delta	34	16	50
	Omicron	4	5	9
Vaccination unavailable		12	51	63
Healthcare worker		3	25	28

When including BMI, sex, and a binary fatigue in the model without depression, the relationship between subjective immune status and cognitive complaints remained significant (*b* = −.496, *p* ≤ .001, R2 change = .020). After correcting for multiple testing using Bonferroni for two regression analyses (0.05/2 = 0.025), this relationship remains statistically significant. Furthermore, depression scores were not observed to account for the relationship between immune status and cognitive failures, as the relationship between immune status and cognitive function remained when a binary depression score was included in model 4 (see Table 4). Finally, to further test the significance of this association, age and education were added as covariates in the model (education proxy was created based on employment title). When including these additional covariates, the effect of ISQ on CFQ remained significant (*b* = −.46, *p* ≤ .001).

**TABLE 4:** Regression analyses with cognitive failures questionnaire (CFQ) scores as outcome.

		B (standardized)	P	R2	R2 Change
1	ISQ	−0.55	<.001	0.305	
					
2	ISQ	−0.548	<.001		0.018
	BMI	−.058	.57		
	Sex	0.12	.26		
					
3	ISQ	−0.5	<.001		0.020
	BMI	−0.76	.45		
	Sex	0.11	.29		
	FACIT‐F	0.15	.16		
					
4	ISQ	−0.45	<.001		0.090
	BMI	−0.08	.37		
	Sex	0.14	.15		
	FACIT‐F	0.05	.65		
	HADS‐D	0.33	.002		

*Note*: Analysis carried out in PCS group only (*n* = 71), after main effects of group and ISQ were detected in whole sample. Model 1: Predictor: ISQ; Model 2: ISQ, sex, BMI; Model 3: ISQ, sex, BMI, FACIT‐F (binary); Model 4: ISQ, sex, BMI, HADS depression (binary).

Abbreviations: BMI, body mass index; CFQ, cognitive failures questionnaire;

FACIT‐F, Functional Assessment of Chronic Illness Therapy‐Fatigue Scale (binary); HADS‐D, hospital anxiety and depression scale, depression score (binary); ISQ, immune status questionnaire; PCS, post‐COVID syndrome group.

## 7 DISCUSSION

The aim of this study was to examine the relationship between self‐reported immune status and subjective cognitive functioning, while exploring whether any observed relationship was accounted for by variation in depression scores. The sample included a PCS group recruited from a hospital “Long Covid” clinic, and a CC group who did not develop long term difficulties following COVID‐19 infection. Using an online questionnaire, we quantified immune status using the immune status Questionnaire (ISQ), and cognitive difficulties were measured based on self‐report using the cognitive failures questionnaire (CFQ). Depression was measured using the Hospital Anxiety and Depression Scale (HADS‐D). Other factors associated with impairment in PCS were also explored in relation to cognitive difficulties, namely, BMI, biological sex, and fatigue, as this is the most common symptom in PCS, and may affect cognitive functioning (Ceban et al., 2022).

In the present study, groups were compared on demographics and key variables of immune status, cognitive failures, mood and fatigue. Although age and sex matched, significant differences between the PCS group and CC were found for ratings of immune status, cognitive functioning, depression, anxiety, and fatigue, with the PCS group having higher levels of symptoms on all measures. These findings are consistent with other studies of PCS, where those with the condition are reported to experience lower immune status and more circulating inflammatory markers in the blood (Kruger et al., 2022), mood difficulties (Benedetti et al., 2021), and fatigue (Guo et al., 2022). Groups did not differ as regards history of anxiety or history of depression, although this has been cited as a factor affecting likelihood of developing PCS in other studies (Hartung et al., 2022). Groups differed significantly regarding date of COVID‐19 infection: participants from a hospital setting for the “PCS” group were mainly infected with COVID‐19 during first wave of the pandemic in Ireland, when vaccination was not available and variants of the virus may have been more neuroinvasive (Bauer et al., 2022). On the other hand, controls were more often infected after the summer of 2021. Due to this difference between groups, along with a high representation of healthcare workers in the sample, study participants may represent a special group of persistent post‐COVID‐19. Although the available control group may not be ideal as a comparison due to these circumstances, study of this group is warranted in an Irish context to further understand PCS. The findings of this study suggest that a combination of self‐reported immune changes, subjective cognitive changes, and mood difficulties may contribute to PCS presentations in an Irish clinical setting.

Findings suggest that self‐reported immune status explains a significant proportion of variance in subjective cognition in the PCS group, even when factoring in changes in mood or fatigue. Considering previous research regarding elevated immune response and cognitive deficits in PCS, this finding was in line with expected results (Costas‐Carrera et al., 2022; de Paula et al., 2022). When including depression, fatigue, sex and BMI, age, and a proxy for education in the model, the relationship between immune status and cognition remained significant. This finding helps to further establish a link between self‐reported changes in immune status and symptoms of PCS, in line with studies of PCS propose that both depression and cognitive function might be linked to underlying inflammatory response (Ferrando et al., 2022; Mazza et al., 2021). Furthermore, when looking at differences between PCS and CCs, the PCS group reported more immune events overall, with the group mean falling above the cut‐off for poor or compromised immune status. This could have implications for clinical practice, as it suggests an immune compromise and physical health impact on subjective cognitive functioning rather than depression and psychological factors only. Further study of the relationship between subjective immune status, cognition, and depression in PCS could help to tailor interventions to improve quality of life for those with PCS.

This study has several limitations. First, although few studies link subjective cognitive, mood, and immune status variables in SARS‐CoV‐2 thus far, our sample of 71 in the PCS “clinical” group could be considered small. Therefore, this analysis needs to be replicated in a larger sample to establish generalizability in the PCS population. Sample sizes in analysis of between group differences were uneven. In contrast to studies with no control group, inclusion of a control group improves the internal validity or attribution of intervention effect. However, having a smaller control group may confound between group differences and increase likelihood of false positives. To reduce this confound, Bonferroni correction was carried out on between group differences results. Second, the measure of immune status used in this study is based on self‐report, without objective measures of immune response. Although previous research has shown significant correlations between self‐rated health and immune biomarkers in both patients (Lekander et al., 2004) and healthy volunteers (Christian et al., 2011; Leshem‐Rubinow et al., 2015; Nakata et al., 2010), some studies suggest that perception of immune functioning was unrelated to immune biomarkers, such as serum antibodies and blood lymphocytes (Petrie et al., 1999). Third, we used a subjective and self‐report measure of cognition, which is also influenced by individual perceptions of functioning, mood, or fatigue (Thames et al., 2011), as opposed to objective measures of cognitive ability. This was due to limited access to participants for face‐to‐face cognitive testing during lockdown. To counter the effect of self‐report, potentially confounding variables such as depression and fatigue were addressed in the regression analysis. Fourth, menopause status was not assessed for this study, and therefore cannot be considered in analyses, although this may be a major factor contributing to PCS presentation. Fifth, as temporality of variables in this analysis cannot be established, it is hard to conclude whether depression symptoms were a cause or consequence of cognitive difficulties, and therefore a causal relationship between the variables is difficult to establish. In order to address this limitation, exploratory analysis comparing mean CFQ scores between those who had a history of mental health difficulties and cognitive failures revealed no significant difference based on mental health history. These findings suggest that cognitive difficulties and depression may be some of many manifestations of PCS in the current population, which are not explained by medical history.

## 8 CONCLUSION

We conducted an analysis to explore the relationship between self‐reported immune status and subjective cognitive functioning in PCS. Further, this study examines whether depression scores account for the relationship between self‐reported immune status and subjective cognition. Our results suggest that self‐reported immune status influences subjective cognition, even when accounting for depression scores in a sample of individuals seeking care for PCS symptoms. These findings could be of importance for the future management of patients, as well as for understanding the emergence of PCS. In clinical practice, the number of self‐reported immune events could be a useful marker of subjective experience in those seeking care for PCS. These findings encourage a more holistic view of functioning in PCS and point to a need for further study of subjective immune response and its impact on subjective cognitive function in this population.

## AUTHOR CONTRIBUTIONS

**Jessica Holland**: Conceptualization; methodology; investigation; writing—review and editing; writing—original draft. **Sinead Brown**: Visualization; project administration; investigation. **Susan O'Flanagan**: Supervision; project administration; data curation; investigation; writing—review and editing. **Stefano Savinelli**: Conceptualization; investigation; data curation; writing—review and editing. **Kathleen McCann**: Investigation; data curation. **Keith Gaynor**: Conceptualization; methodology; data curation. **Patrick Mallon**: Investigation; resources. **Eoin Feeney**: Investigation; resources. **Grace Kenny**: Investigation; resources. **Christine Boyd**: Software. **Fiadhnait O'Keeffe**: Supervision; data curation; conceptualization; writing—review and editing. **Jessica Bramham**: Supervision; conceptualization; data curation; formal analysis; validation; resources; writing—review and editing.

## CONFLICT OF INTEREST STATEMENT

The authors declare no conflicts of interest.

### PEER REVIEW

The peer review history for this article is available at https://publons.com/publon/10.1002/brb3.70027.

## Data Availability

The data that support the findings of this study are available from the corresponding author upon reasonable request.

## References

[brb370027-bib-0001] Almeria, M. , Cejudo, J. C. , Sotoca, J. , Deus, J. , & Krupinski, J. (2020). Cognitive profile following COVID‐19 infection: Clinical predictors leading to neuropsychological impairment. Brain, Behavior, & Immunity‐Health, 9, 100163. 10.1016/j.bbih.2020.100163 PMC7581383 33111132

[brb370027-bib-0003] Bauer, L. , Rissmann, M. , Benavides, F. F. W. , Leijten, L. , Van Run, P. , Begeman, L. , Veldhuis Kroeze, E. J. B. , Lendemeijer, B. , Smeenk, H. , De Vrij, F. M. S. , Kushner, S. A. , Koopmans, M. P. G. , Rockx, B. , & Van Riel, D. (2022). In vitro and in vivo differences in neurovirulence between D614G, delta and omicron BA. 1 SARS‐CoV‐2 variants. Acta Neuropathologica Communications, 10(1), 124. 10.1186/s40478-022-01426-4 36058935 PMC9441226

[brb370027-bib-0004] Benedetti, F. , Mazza, M. , Cavalli, G. , Ciceri, F. , Dagna, L. , & Rovere‐Querini, P. (2021). Can cytokine blocking prevent depression in COVID‐19 survivors? Journal of Neuroimmune Pharmacology, 16, 1–3. 10.1007/s11481-020-09966-z 33107012 PMC7587545

[brb370027-bib-0005] Bower, J. E. , Radin, A. , & Kuhlman, K. R. (2022). Psychoneuroimmunology in the time of COVID‐19: Why neuro‐immune interactions matter for mental and physical health. Behaviour Research and Therapy, 154, 104104. 10.1016/j.brat.2022.104104 35609375 PMC9075982

[brb370027-bib-0006] Bridger, R. S. , Johnsen, S. Å. K. , & Brasher, K. (2013). Psychometric properties of the cognitive failures questionnaire. Ergonomics, 56(10), 1515–1524. 10.1080/00140139.2013.821172 23879800

[brb370027-bib-0007] Broadbent, D. E. , Cooper, P. F. , Fitzgerald, P. , & Parkes, K. R. (1982). The cognitive failures questionnaire (CFQ) and its correlates. British Journal of Clinical Psychology, 21(1), 1–16. 10.1111/j.2044-8260.1982.tb01421.x 7126941

[brb370027-bib-0008] Carod Artal, F. J. (2021). Post‐COVID‐19 syndrome: Epidemiology, diagnostic criteria and pathogenic mechanisms involved. Revista de neurologia, 72(11), 384–396. 10.33588/rn.7211.2021230 34042167

[brb370027-bib-0009] Ceban, F. , Ling, S. , Lui, L. M. W. , Lee, Y. , Gill, H. , Teopiz, K M. , Rodrigues, N. B. , Subramaniapillai, M. , Di Vincenzo, J. D. , Cao, B. , Lin, K. , Mansur, R. B. , Ho, R. C. , Rosenblat, J. D. , Miskowiak, K. W. , Vinberg, M. , Maletic, V. , & Mcintyre, R. S. (2022). Fatigue and cognitive impairment in Post‐COVID‐19 Syndrome: A systematic review and meta‐analysis. Brain, Behavior, and Immunity, 101, 93–135. 10.1016/j.bbi.2021.12.020 34973396 PMC8715665

[brb370027-bib-0010] Ceban, F. , Nogo, D. , Carvalho, I. P. , Lee, Y. , Nasri, F. , Xiong, J. , Lui, L. M. W. , Subramaniapillai, M. , Gill, H. , Liu, R. N. , Joseph, P. , Teopiz, K. M. , Cao, B. , Mansur, R. B. , Lin, K. , Rosenblat, J. D. , Ho, R. C. , & Mcintyre, R. S. (2021). Association between mood disorders and risk of COVID‐19 infection, hospitalization, and death: A systematic review and meta‐analysis. JAMA Psychiatry, 78(10), 1079–1091. 10.1001/jamapsychiatry.2021.1818 34319365 PMC8319830

[brb370027-bib-0011] Cella, D. , Lai, J.‐S. , Chang, C.‐H. , Peterman, A. , & Slavin, M. (2002). Fatigue in cancer patients compared with fatigue in the general United States population. Cancer, 94(2), 528–538. 10.1002/cncr.10245 11900238

[brb370027-bib-0012] Christian, L. M. , Glaser, R. , Porter, K. , Malarkey, W. B. , Beversdorf, D. , & Kiecolt‐Glaser, J. K. (2011). Poorer self‐rated health is associated with elevated inflammatory markers among older adults. Psychoneuroendocrinology, 36(10), 1495–1504. 10.1016/j.psyneuen.2011.04.003 21601365 PMC3161147

[brb370027-bib-0014] Costas‐Carrera, A. , Sánchez‐Rodríguez, M. M. , Cañizares, S. , Ojeda, A. , Martín‐Villalba, I. , Primé‐Tous, M. , Rodríguez‐Rey, M. A. , Segú, X. , Valdesoiro‐Pulido, F. , Borras, R. , Peri, J. M. , & Vieta, E. (2022). Neuropsychological functioning in post‐ICU patients after severe COVID‐19 infection: The role of cognitive reserve. Brain Behavior, & Immunity—Health, 21, 100425. 10.1016/j.bbih.2022.100425 PMC8818554 35156065

[brb370027-bib-0015] Davis, H. E. , McCorkell, L. , Vogel, J. M. , & Topol, E. J. (2023). Long COVID: Major findings, mechanisms and recommendations. Nature Reviews Microbiology, 21, 133–146. 36639608 10.1038/s41579-022-00846-2 PMC9839201

[brb370027-bib-0016] de Paula, J. J. , Paiva, R. E. R. P. , Souza‐Silva, N. G. , Rosa, D. V. , Duran, F. L. d. S. , Coimbra, R. S. , de Souza Costa, D. , Dutenhefner, P. R. , Oliveira, H. S. D. , Camargos, S. T. , Vasconcelos, H. M. M. , de Oliveira Carvalho, N. , da Silva, J. B. , Silveira, M. B. , Malamut, C. , Oliveira, D. M. , Molinari, L. C. , de Oliveira, D. B. , Januário, J. N. , … Romano‐Silva, M. A. (2022). Selective visuoconstructional impairment following mild covid‐19 with inflammatory and neuroimaging correlation findings. Molecular Psychiatry, 28, 553–563. 35701598 10.1038/s41380-022-01632-5 PMC9196149

[brb370027-bib-0019] Evans, R. A. , Mcauley, H. J. C. , Harrison, E. M. , Shikotra, A. , Singapuri, A. , Sereno, M. , Elneima, O. , Docherty, A. B. , Lone, N. I. , Leavy, O. C. , Daines, L. , Baillie, J. K. , Brown, J. S. , Chalder, T. , De Soyza, A. , Diar Bakerly, N. , Easom, N. , Geddes, J. R. , Greening, N. J. , … Zongo, O. (2021). Physical, cognitive, and mental health impacts of COVID‐19 after hospitalisation (PHOSP‐COVID): A UK multicentre, prospective cohort study. The Lancet Respiratory Medicine, 9(11), 1275–1287. 10.1016/S2213-2600(21)00383-0 34627560 PMC8497028

[brb370027-bib-0020] Faul, F. , Erdfelder, E. , Lang, A.‐G. , & Buchner, A. (2007). G* Power 3: A flexible statistical power analysis program for the social, behavioral, and biomedical sciences. Behavior Research Methods, 39(2), 175–191. 10.3758/BF03193146 17695343

[brb370027-bib-0021] Fernández‐De‐Las‐Peñas, C. , Rodríguez‐Jiménez, J. , Palacios‐Ceña, M. , De‐La‐Llave‐Rincón, A. I. , Fuensalida‐Novo, S. , Florencio, L L. , Ambite‐Quesada, S. , Ortega‐Santiago, R. , Arias‐Buría, J. L. , Liew, B. X. W. , Hernández‐Barrera, V. , & Cigarán‐Méndez, M. (2022). Psychometric properties of the hospital anxiety and depression scale (HADS) in previously hospitalized COVID‐19 patients. International Journal of Environmental Research and Public Health, 19(15), 9273. 10.3390/ijerph19159273 35954630 PMC9367824

[brb370027-bib-0022] Ferrando, S. J. , Dornbush, R. , Lynch, S. , Shahar, S. , Klepacz, L. , Karmen, C. L. , Chen, D. , Lobo, S. A. , & Lerman, D. (2022). Neuropsychological, medical, and psychiatric findings after recovery from acute COVID‐19: A cross‐sectional study. Journal of the Academy of Consultation—Liaison Psychiatry, 63(5), 474–484. 10.1016/j.jaclp.2022.01.003 35085824 PMC8786396

[brb370027-bib-0023] Ferrucci, R. , Dini, M. , Groppo, E. , Rosci, C. , Reitano, M. R. , Bai, F. , Poletti, B. , Brugnera, A. , Silani, V. , D'arminio Monforte, A. , & Priori, A. (2021). Long‐lasting cognitive abnormalities after COVID‐19. Brain Sciences, 11(2), 235. 10.3390/brainsci11020235 33668456 PMC7917789

[brb370027-bib-0027] Guo, P. Y. , Ballesteros, A. B. , Yeung, S. P. , Liu, R. B. Y. , Saha, A. , Curtis, L. , Kaser, M. , Haggard, M. P., & Cheke, L. G. (2022). COVCOG 1: Factors predicting physical, neurological and cognitive symptoms in long COVID in a community sample. A first publication from the COVID and cognition study. Frontiers In Aging Neuroscience, 14, 804922. 35370617 10.3389/fnagi.2022.804922 PMC8968323

[brb370027-bib-0028] Hartung, T J. , Neumann, C. , Bahmer, T. , Chaplinskaya‐Sobol, I. , Endres, M. , Geritz, J. , Haeusler, K. G. , Heuschmann, P. U. , Hildesheim, H. , Hinz, A. , Hopff, S. , Horn, A. , Krawczak, M. , Krist, L. , Kudelka, J. , Lieb, W. , Maetzler, C. , Mehnert‐Theuerkauf, A. , Montellano, F A. , … Finke, C. (2022). Fatigue and cognitive impairment after COVID‐19: A prospective multicentre study. EClinicalMedicine, 53, 101651. 10.1016/j.eclinm.2022.101651 36133318 PMC9482331

[brb370027-bib-0029] Helms, J. , Kremer, S. , Merdji, H. , Clere‐Jehl, R. , Schenck, M. , Kummerlen, C. , Collange, O. , Boulay, C. , Fafi‐Kremer, S. , Ohana, M. , Anheim, M. , & Meziani, F. (2020). Neurologic features in severe SARS‐CoV‐2 infection. New England Journal of Medicine, 382(23), 2268–2270. 10.1056/NEJMc2008597 32294339 PMC7179967

[brb370027-bib-0030] Heneka, M. T. , Golenbock, D. , Latz, E. , Morgan, D. , & Brown, R. (2020). Immediate and long‐term consequences of COVID‐19 infections for the development of neurological disease. Alzheimer's Research & Therapy, 12, 1–3. 10.1186/s13195-020-00640-3 PMC7271826 32498691

[brb370027-bib-0031] Hopkins, R. O. , Weaver, L. K. , Pope, D. , Orme, J. F. , Bigler, E. D. , & Larson‐Lohr, V. (1999). Neuropsychological sequelae and impaired health status in survivors of severe acute respiratory distress syndrome. American Journal of Respiratory and Critical Care Medicine, 160(1), 50–56. 10.1164/ajrccm.160.1.9708059 10390379

[brb370027-bib-0032] Hu, B. , Huang, S. , & Yin, L. (2021). The cytokine storm and COVID‐19. Journal of Medical Virology, 93(1), 250–256. 10.1002/jmv.26232 32592501 PMC7361342

[brb370027-bib-0033] Huang, C. , Huang, L. , Wang, Y. , Li, X. , Ren, L. , Gu, X. , Kang, L. , Guo, L. i. , Liu, M. , Zhou, X. , Luo, J. , Huang, Z. , Tu, S. , Zhao, Y. , Chen, L. i. , Xu, D. , Li, Y. , Li, C. , Peng, L. u. , … Cao, B. (2021). 6‐month consequences of COVID‐19 in patients discharged from hospital: A cohort study. The Lancet, 397(10270), 220–232. 10.1016/S0140-6736(20)32656-8 PMC7833295 33428867

[brb370027-bib-0034] Huang, C.‐Y. , Huang, W.‐H. , & Yen, H.‐Y. (2023). An exploration of sedentary behavior, physical activity, and quality of life during the COVID‐19 outbreak. International Journal of Public Health, 68, 1605585. 36776738 10.3389/ijph.2023.1605585 PMC9908611

[brb370027-bib-0037] Kruger, A. , Vlok, M. , Turner, S. , Venter, C. , Laubscher, G. J. , Kell, D. B. , & Pretorius, E. (2022). Proteomics of fibrin amyloid microclots in long COVID/post‐acute sequelae of COVID‐19 (PASC) shows many entrapped pro‐inflammatory molecules that may also contribute to a failed fibrinolytic system. Cardiovasc Diabetol, 21(1), 190. 10.1186/s12933-022-01623-4 36131342 PMC9491257

[brb370027-bib-0039] Lekander, M. , Elofsson, S. , Neve, I.‐M. , Hansson, L.‐O. , & Undén, A.‐L. (2004). Self‐rated health is related to levels of circulating cytokines. Psychosomatic Medicine, 66(4), 559–563. 10.1097/01.psy.0000130491.95823.94 15272103

[brb370027-bib-0040] Leshem‐Rubinow, E. , Shenhar‐Tsarfaty, S. , Milwidsky, A. , Toker, S. , Shapira, I. , Berliner, S. , Benyamini, Y. , Melamed, S. , & Rogowski, O. (2015). Self‐rated health is associated with elevated C‐reactive protein even among apparently healthy individuals. The Israel Medical Association Journal: IMAJ, 17(4), 213–218. 26040045

[brb370027-bib-0041] Lier, J. , Stoll, K. , Obrig, H. , Baum, P. , Deterding, L. , Bernsdorff, N. , Hermsdorf, F. , Kunis, I. , Bräsecke, A. , Herzig, S. , Schroeter, M. L. , Thöne‐Otto, A. , Riedel‐Heller, S. G. , Laufs, U. , Wirtz, H. , Classen, J. , & Saur, D. (2022). Neuropsychiatric phenotype of post COVID‐19 syndrome in non‐hospitalized patients. Frontiers in Neurology, 13, 988359. 36237627 10.3389/fneur.2022.988359 PMC9552839

[brb370027-bib-0042] Machado, F. V. C. , Meys, R. , Delbressine, J. M. , Vaes, A. W. , Goërtz, Y. M. J. , Van Herck, M. , Houben‐Wilke, S. , Boon, G. J. A. M. , Barco, S. , Burtin, C. , Van ’T Hul, A. , Posthuma, R. , Franssen, F. M. E. , Spies, Y. , Vijlbrief, H. , Pitta, F. , Rezek, S. A. , Janssen, D. J. A. , Siegerink, B. , … Spruit, M. A. (2021). Construct validity of the Post‐COVID‐19 functional status scale in adult subjects with COVID‐19. Health and Quality of Life Outcomes, 19, 1–10. 10.1186/s12955-021-01691-2 33536042 PMC7856622

[brb370027-bib-0043] Mazza, M. G. , De Lorenzo, R. , Conte, C. , Poletti, S. , Vai, B. , Bollettini, I. , Melloni, E. M. T. , Furlan, R. , Ciceri, F. , Rovere‐Querini, P. , & Benedetti, F. (2020). Anxiety and depression in COVID‐19 survivors: Role of inflammatory and clinical predictors. Brain, Behavior, and Immunity, 89, 594–600. 10.1016/j.bbi.2020.07.037 32738287 PMC7390748

[brb370027-bib-0044] Mazza, M. G. , Palladini, M. , De Lorenzo, R. , Magnaghi, C. , Poletti, S. , Furlan, R. , Ciceri, F. , Rovere‐Querini, P. , & Benedetti, F. (2021). Persistent psychopathology and neurocognitive impairment in COVID‐19 survivors: Effect of inflammatory biomarkers at three‐month follow‐up. Brain, Behavior, and Immunity, 94, 138–147. 10.1016/j.bbi.2021.02.021 33639239 PMC7903920

[brb370027-bib-0045] Miskowiak, K. , Johnsen, S. , Sattler, S. , Nielsen, S. , Kunalan, K. , Rungby, J. , Lapperre, T. , & Porsberg, C. (2021). Cognitive impairments four months after COVID‐19 hospital discharge: Pattern, severity and association with illness variables. European Neuropsychopharmacology, 46, 39–48. 10.1016/j.euroneuro.2021.03.019 33823427 PMC8006192

[brb370027-bib-0046] Montan, I. , Löwe, B. , Cella, D. , Mehnert, A. , & Hinz, A. (2018). General population norms for the functional assessment of chronic illness therapy (FACIT‐F)‐fatigue scale. Value in Health, 21(11), 1313–1321. 10.1016/j.jval.2018.03.013 30442279

[brb370027-bib-0047] Méndez, R. , Balanzá‐Martínez, V. , Luperdi, S. C. , Estrada, I. , Latorre, A. , González‐Jiménez, P. , Bouzas, L. , Yépez, K. , Ferrando, A. , Reyes, S. , & Menéndez, R. (2022). Long‐term neuropsychiatric outcomes in COVID‐19 survivors: A 1‐year longitudinal study. Journal of Internal Medicine, 291(2), 247. 10.1111/joim.13389 34569681 PMC8662064

[brb370027-bib-0048] Nakata, A. , Takahashi, M. , Otsuka, Y. , & Swanson, N. G. (2010). Is self‐rated health associated with blood immune markers in healthy individuals? International Journal of Behavioral Medicine, 17, 234–242. 10.1007/s12529-010-9102-0 20512441

[brb370027-bib-0049] Olssøn, I. , Mykletun, A. , & Dahl, A. A. (2005). The hospital anxiety and depression rating scale: A cross‐sectional study of psychometrics and case finding abilities in general practice. BMC Psychiatry, 5, 1–7. 10.1186/1471-244X-5-46 16351733 PMC1343544

[brb370027-bib-0050] Petrie, K. J. , Booth, R. J. , Elder, H. , & Cameron, L. D. (1999). Psychological influences on the perception of immune function. Psychological Medicine, 29(2), 391–397. 10.1017/S003329179800782X 10218929

[brb370027-bib-0051] Piper, B. F. , & Cella, D. (2010). Cancer‐related fatigue: Definitions and clinical subtypes. Journal of the National Comprehensive Cancer Network, 8(8), 958–966. 10.6004/jnccn.2010.0070 20870639

[brb370027-bib-0052] Poletti, S. , Palladini, M. , Mazza, M. G. , De Lorenzo, R. , Furlan, R. , Ciceri, F. , Rovere‐Querini, P. , & Benedetti, F. (2021). Long‐term consequences of COVID‐19 on cognitive functioning up to 6 months after discharge: Role of depression and impact on quality of life. European Archives of Psychiatry and Clinical Neuroscience, 272, 773–782. 34698871 10.1007/s00406-021-01346-9 PMC8546751

[brb370027-bib-0053] Rosenblat, J. D. , Brietzke, E. , Mansur, R. B. , Maruschak, N. A. , Lee, Y. , & Mcintyre, R. S. (2015). Inflammation as a neurobiological substrate of cognitive impairment in bipolar disorder: Evidence, pathophysiology and treatment implications. Journal of Affective Disorders, 188, 149–159. 10.1016/j.jad.2015.08.058 26363613

[brb370027-bib-0054] Sheng, B. , Wing Cheng, S. K. , Lau, K. K. , Li, H. L. , & Yiu Chan, E. L. (2005). The effects of disease severity, use of corticosteroids and social factors on neuropsychiatric complaints in severe acute respiratory syndrome (SARS) patients at acute and convalescent phases. European Psychiatry, 20(3), 236–242. 10.1016/j.eurpsy.2004.06.023 15935422 PMC7135192

[brb370027-bib-0055] Taquet, M. , Geddes, J. R. , Husain, M. , Luciano, S. , & Harrison, P. J. (2021). 6‐month neurological and psychiatric outcomes in 236 379 survivors of COVID‐19: A retrospective cohort study using electronic health records. The Lancet Psychiatry, 8(5), 416–427. 10.1016/S2215-0366(21)00084-5 33836148 PMC8023694

[brb370027-bib-0056] Thames, A. D. , Becker, B. W. , Marcotte, T. D. , Hines, L. J. , Foley, J. M. , Ramezani, A. , Singer, E. J. , Castellon, S. A. , Heaton, R. K. , & Hinkin, C. H. (2011). Depression, cognition, and self‐appraisal of functional abilities in HIV: An examination of subjective appraisal versus objective performance. The Clinical Neuropsychologist, 25(2), 224–243. 10.1080/13854046.2010.539577 21331979 PMC3616492

[brb370027-bib-0057] Wilod Versprille, L. J. F. , Van De Loo, A. J. A. E. , Mackus, M. , Arnoldy, L. , Sulzer, T. A. L. , Vermeulen, S. , Abdulahad, S. , Huls, H. , Baars, T. , Scholey, A. , Kraneveld, A. , Garssen, J. , & Verster, J. (2019). Development and validation of the immune status questionnaire (ISQ). International Journal of Environmental Research and Public Health, 16(23), 4743. 10.3390/ijerph16234743 31783555 PMC6926937

[brb370027-bib-0059] World Health Organization . (2021). *A clinical case definition of post COVID‐19 condition by a Delphi consensus* (No. WHO/2019‐nCoV/Post_COVID‐19_condition/Clinical_case_definition/2021.1). World Health Organization.

[brb370027-bib-0060] Yellen, S. B. , Cella, D. F. , Webster, K. , Blendowski, C. , & Kaplan, E. (1997). Measuring fatigue and other anemia‐related symptoms with the functional assessment of cancer therapy (FACT) measurement system. Journal of Pain and Symptom Management, 13(2), 63–74. 10.1016/S0885-3924(96)00274-6 9095563

[brb370027-bib-0061] Zalli, A. , Jovanova, O. , Hoogendijk, W. J. G. , Tiemeier, H. , & Carvalho, L. A. (2016). Low‐grade inflammation predicts persistence of depressive symptoms. Psychopharmacology, 233(9), 1669–1678. 10.1007/s00213-015-3919-9 25877654 PMC4828485

[brb370027-bib-0062] Zhou, H. , Lu, S. , Chen, J. , Wei, N. , Wang, D. , Lyu, H. , Shi, C. , & Hu, S. (2020). The landscape of cognitive function in recovered COVID‐19 patients. Journal of Psychiatric Research, 129, 98–102. 10.1016/j.jpsychires.2020.06.022 32912598 PMC7324344

